# Hyperhomocysteinemia and Lupus Nephritis

**DOI:** 10.7759/cureus.5065

**Published:** 2019-07-02

**Authors:** Homa Timlin, Rebecca Manno, Homeyra Douglas

**Affiliations:** 1 Rheumatology, The Johns Hopkins University School of Medicine, Baltimore, USA; 2 Cardiology, Aintree University Hospital, Liverpool, GBR

**Keywords:** thrombosis, lupus nephritis, cardiovascular diseases, coronary artery disease, cardiovascular disease, hyperhomocysteinemia, atherosclerosis, lupus

## Abstract

Background

In SLE, both disease-specific and traditional risk factors are important. Increased serum homocysteine levels are seen in approximately 15% of patients with systemic lupus erythematosus and are associated with an increased risk of atherothrombotic events in this population. The serum level of homocysteine in patients with lupus nephritis has not been well described.

Methods

We performed a retrospective review of patients who had both biopsy-proven lupus nephritis (class II-VI) and measured homocysteine levels during routine evaluation. Clinical and laboratory data were obtained from reviews of medical records.

Results

Of the 15 patients with lupus nephritis, 10 had elevated homocysteine levels. The ages ranged from 21-68 years and were predominately African-American females. There were three patients with class III, one with class III-V, two with class IV, and two with class V lupus nephritis. Two patients had more than one biopsy each, one with class III, IV-V, and one with III and IV. At the time, when the serum homocysteine level was measured, of the 10 patients with elevated homocysteine levels, five patients had positive anti-dsDNA, and four had hypocomplementemia predominately low C3 (three patients). All patients were on hydroxychloroquine.

Conclusions

This study demonstrates that patients with lupus nephritis are at a higher risk (66.6%) for developing elevated homocysteine levels.

## Introduction

Homocysteine is metabolized by two alternative pathways, including its remethylation and transsulfuration. The remethylation pathway regenerates methionine by methylenetetrahydrofolate reductase (MTHFR) using homocysteine as substrate and folate and vitamin B as cofactors [1-4]. Elevations in plasma homocysteine levels can result from genetic factors such as methylene tetrahydrofolate reductase (MTHFR) mutation, vitamin deficiency (specifically deficiency of folate, vitamin B6, or vitamin B12), and chronic kidney disease.

Elevated serum homocysteine can occur in 5 to 10 percent of the population [5]. Majority of studies have shown that elevated serum levels of homocysteine have primary atherogenic and prothrombotic properties. Hyperhomocysteinemia has also been recognized as a pathogenic factor in the progression of end-stage renal disease. Furthermore, high homocysteine levels in adults have been associated with lower extremity peripheral arterial disease, heart failure, osteoporosis fractures, dementia, and cognitive impairment [6-11].

Correcting nutritional inadequacy of folic acid and vitamin B will lower homocysteine levels in most patients [12]. 

Increased serum homocysteine levels are seen in approximately 15% of patients with systemic lupus erythematosus. We postulated that patients with lupus nephritis are more likely to have elevated homocysteine levels.

## Materials and methods

In this retrospective study, patients were included if they had biopsy-proven lupus nephritis and available homocysteine levels. This study was approved by the Johns Hopkins University School of Medicine Institutional Review Board. Raised homocysteine concentrations were defined as more than 12 umol/L (normal lab value 0-12.2 umol/l). Anti-double stranded DNA was considered positive if the value was above twice the reference range. Renal function was estimated using the four-variable modification of diet in renal disease (MDRD) formula for estimated GFR (e-GFR). Proteinuria was defined by lab value with a urine protein:creatinine ratio of >0.19 gm/g Cr. Clinical and laboratory data were obtained from the review of medical records.

## Results

In total, 15 patients with biopsy-proven lupus nephritis had measured homocysteine level. At the time, when the serum homocysteine level was measured, eight patients had positive anti-dsDNA (53%) and six had hypocomplementemia (40%). Urine protein:creatinine ratio was elevated in eight (53%), and eGFR was low in two patients (13%). Of the fifteen, ten patients had elevated homocysteine level (66%). Their ages ranged from 21-68 years and were predominately African-American females (90%; Table 1).

**Table 1:** Demographics and serologic features at the time the level of homocysteine was measured M = male; F = female; AA = African-American; anti-dsDNA = anti-double-stranded DNA, positive if above twice the reference range; Y = yes; N = normal; H = homogenous; S = speckled; C = Complement

Subject number	Age	Gender	Race	C3	C4	ANA	dsDNA
1	29	F	AA	N	Low	>640 S	Y (1:40)
2	30	F	AA	Low	N	>640 S	Y (1:20)
3	30	F	AA	N	N	>640 S	Y (1:160)
4	40	F	AA	Low	N	>1280 S	Y (>640)
5	68	F	AA	N	N	320	Negative
6	47	F	Other	Low	Low	>640 S	Negative
7	45	M	AA	N	N	>640 H	Negative
8	21	F	AA	N	N	640 S	Negative
9	49	F	AA	N	N	1280 S	Negative
10	31	F	AA	N	N	>640 S	Y (1:10)

There were three patients with class III, one with class III-V, two with class IV, and two with class V lupus nephritis. Two patients had more than one biopsy, one of them with class III, IV-V, and one with III and IV. Five patients had positive anti-dsDNA (50%), and four had hypocomplementemia predominately C3 (three patients). Approximately 80% had elevated urine protein creatinine ratio (0.23-5.7 gm/g Cr), and eGFR was low in two patients (20%). One patient had albumin below 3.5 g/dl (Table 2).

**Table 2:** Serologic features at the time the level of homocysteine was measured N = normal; eGFR = estimated glomerular filtration rate; N = normal; UPCR = urine protein:creatinine ratio

Subject number	Homocysteine >12 umol/l (normal: 0-12.2umol/l)	eGFR (normal: >60 mL/min/1.73 sqm)	albumin (normal value: 3.5 - 5.3 g/dL)	UPCR (normal: 0.00-0.19)
1	13.9	N	4.2	0.36
2	12.1	N	4.3	0.41
3	16.8	N	3.9	1.9
4	27.7	Low (34)	3.1	5.7
5	18.6	N	4.3	0.34
6	12.9	N	4.1	0.3
7	13.8	N	4.8	N (0.15)
8	15.7	N	4.2	0.23
9	16.7	N	4.5	N (0.13)
10	244.3	Low (9)	3.6	0.81

All 10 patients were on hydroxychloroquine, six on prednisone (60%), and seven on mycophenolate (70%; Table 3).

**Table 3:** Medications at the time the level of homocysteine was measured Y = yes; N = no; ACEi = angiotensin-converting enzyme inhibitor; ARB = angiotensin receptor blocker; MMF = mycophenolate/myfortic; RTX = rituximab

Subject Number	Steroids	ACEi/ARB	Hydroxychloroquine	MMF/Myfortic	Tacrolimus	Biologics
1	Y	Y	Y	Y	Y	N
2	N	N	Y	Y	N	RTX/Benlysta
3	Y	Y	Y	Y	N	N
4	N	N	Y	Y	N	N
5	Y	N	Y	Y	N	N
6	Y	Y	Y	Y	N	N
7	N	Y	Y	N	N	N
8	N	Y	Y	Y	N	N
9	Y	N	Y	N	N	N
10	Y	N	Y	N	N	N

## Discussion

Identification of disease-related risk factors for accelerated atherosclerosis in SLE will be essential for the classification of high-risk subjects to allow for more effective trial designs and the discovery of preventive strategies. The association of elevated homocysteine with cardiovascular disease and stroke was illustrated by a meta-analysis that evaluated data from 12 prospective studies, involving 1855 coronary heart disease (CHD) events and 463 stroke events [4]. Studies have found an association between mild hyperhomocysteinemia and occlusive vascular disease.

Accelerated atherosclerosis is more common in women with systemic lupus erythematosus compared to the general population. Petri et al. found raised homocysteine concentrations in 15% of SLE patients. After adjustment for established risk factors, total plasma homocysteine concentrations remained an independent risk factor for stroke (odds ratio: 2·44) and arterial thromboses (odds ratio: 3·49) [13]. These findings were confirmed by other studies [14-15].

We previously reported an elevated homocysteine level in five patients with lupus nephritis who had measured homocysteine levels during routine visits [16]. Our current study also demonstrates a high association with elevated homocysteine concentrations in patients with lupus nephritis (66%). Moroni et al. found that serum creatinine and active lupus nephritis were independent predictors of hyperhomocysteniemia [17]. We found that at the time, when the serum homocysteine was measured, 50% had positive anti-dsDNA, 40% had hypocomplementemia. Majority (80%) had elevated urine protein creatinine ratio (0.23-5.7 gm/g Cr). Furthermore, only 20% had low eGFR, indicating that patients with lupus nephritis can have elevated serum homocysteine level independent to abnormal renal function. These findings also suggest a possible role of silent inflammation in maintaining an oxidative stress condition. Moreover, Petri et al. found that after treating elevated homocysteine with Folbic or folic acid, there was a 10-µmol/L decrease in homocysteine within an individual that was associated with a 0.011-unit decrease in the urine protein/creatinine ratio.

Although some studies have yet to determine whether lowering homocysteine levels can reduce coronary events, aggressive control of SLE and the lowering of homocysteine concentrations are a potential means to retard the development and progression of atherosclerosis in SLE. Our study has some limitations, such as small sample size, and retrospective analysis.

Further studies are much needed in understanding the mechanisms responsible for elevated homocysteine level in patients with lupus nephritis and further risk stratification criteria in this vulnerable group of patients. 

## Conclusions

Our findings suggest that patients with lupus nephritis are at higher risk of developing hyperhomocysteinemia. Therefore, as a modifiable maker, homocysteine should be measured in all patients with lupus nephritis and treat hyperhomocyteinemia with Folbic (combination of vitamin B and folic acid). 
